# Biparietal diameter *vs* crown–rump length as standard parameter for late first‐trimester pregnancy dating

**DOI:** 10.1002/uog.29124

**Published:** 2024-10-24

**Authors:** H. K. Gjessing, P. Grøttum, J. M. Dreier, S. H. Eik‐Nes

**Affiliations:** ^1^ Centre for Fertility and Health, Norwegian Institute of Public Health Oslo Norway; ^2^ Department of Global Public Health and Primary Care University of Bergen Bergen Norway; ^3^ Section of Medical Informatics University of Oslo Oslo Norway; ^4^ National Center for Fetal Medicine, Department of Obstetrics and Gynecology St Olav's University Hospital Trondheim Norway; ^5^ Department of Laboratory Medicine, Children's and Women's Health Norwegian University of Science and Technology Trondheim Norway

**Keywords:** biparietal diameter, crown–rump length, first trimester, gestational age, population setting, routine ultrasound, term prediction, time‐to‐event analysis

## Abstract

**Objective:**

To compare the precision of biparietal diameter (BPD) and crown–rump length (CRL) as predictors of gestational age in the human fetus in the late first and early second trimesters, using a population‐based approach.

**Methods:**

We constructed term and gestational‐age prediction curves for first‐trimester dating, based on 11 041 pregnancies with 12 260 measurements of CRL and/or BPD from a population‐based Norwegian clinical database. We used a population‐based approach with local linear quantile regression, combined with a time‐to‐event strategy that compensates for induced births. Term prediction precision was assessed by estimating and comparing the prediction residual curves using a time‐to‐event analysis. Individual differences in gestational‐age predictions from CRL and BPD were assessed using measurements performed on the same fetus on the same day. A sensitivity analysis was performed to evaluate the effect of not distinguishing between non‐spontaneous and spontaneous births.

**Results:**

CRL and BPD provided almost identical term prediction precision judged from the residual distribution. In about 51% of examinations, the difference in predicted gestational age was 1 day or less; 24% of examinations had a difference of 2 days, 14% had a difference of 3 days, 7% had a difference of 4 days and only 5% of all examinations had a difference of 5 days or more. Incorrectly removing induced births from the analysis, or treating them as spontaneous, would cause a substantial systematic prediction bias of about 2 days.

**Conclusions:**

Based on population data, using comparisons at an individual level, our study found that BPD is as precise as CRL when used for first‐trimester dating. BPD has advantages from a clinical point of view, since it is technically less challenging and less time‐consuming to measure compared with CRL, and can be measured and assessed throughout the entire pregnancy. © 2024 The Author(s). *Ultrasound in Obstetrics & Gynecology* published by John Wiley & Sons Ltd on behalf of International Society of Ultrasound in Obstetrics and Gynecology.

**CONTRIBUTION**

*What are the novel findings of this work?*

Gestational age and term predictions based on biparietal diameter (BPD) and those based on crown–rump length (CRL) are of almost identical precision when assessed at an individual level in a population setting in the late first trimester.

*What are the clinical implications of this work?*

The measurement of BPD can replace the measurement of CRL for dating the fetus in the late first trimester. BPD measurement is generally less complicated to perform compared with CRL measurement and is used throughout pregnancy.

## INTRODUCTION

Early assessment of the human embryo was initiated by embryologists studying the stage of maturity of aborted embryos. The term crown–rump length (CRL) was introduced in 1907 by Mall at the Anatomical Laboratory at Johns Hopkins University in Baltimore, MD, USA^1^. Since then, the term CRL has been used universally.

In 1973, Robinson was the first to use sonar technology to measure CRL and assess embryological development *in vivo* ^2^. He confirmed his findings by comparing them with post‐abortion measurements of CRL. Together with Fleming, Robinson followed up this research 2 years later with a detailed evaluation of his technique for *in‐vivo* assessment of the fetus, and compared his sonar measurements with embryologists' growth curves (notably those of Mall and Streeter)^3^. As early as 1975, Robinson and Fleming pointed out that ‘a reasonable degree of care and patience is required to achieve accurate and reliable results particularly after the tenth week, when fetal movements may be troublesome’^3^.

The world's first ultrasound screening program for an entire pregnant population was introduced in Malmö, Sweden, in 1973, with the initial purpose of detecting twins. The examination, which was performed at week 28, also revealed important anatomical details about the fetus, so in 1976 it was moved to week 18/19^4^. The 18/19‐week examination also allowed for dating of the pregnancy. This was the start of ultrasound screening in pregnancy.

The improved quality of ultrasound technology has made it possible to assess the anatomy of the fetus in the late first trimester. Using CRL for dating has proved to be precise prior to week 10, but the precision has been shown to decrease over the subsequent 3 weeks^5^, ^6^. As a parameter that is more easily measured and standardized, the biparietal diameter (BPD) may be an alternative for systematic late first‐trimester dating.

The purpose of this study was to compare the precision of gestational age and estimated day of delivery (EDD) prediction by BPD *vs* CRL in the late first trimester. We employed population data and time‐to‐event (TTE) analysis to compensate for pregnancies that were induced.

## METHODS

### Population and variables

Our data, collected continuously from 1987 to 2017, cover the city of Trondheim and eight surrounding municipalities in Norway, thus representing a non‐selected population from a geographically delimited area. All ultrasound examinations were performed using high‐quality ultrasound machines at the National Center for Fetal Medicine at St Olav's University Hospital, Trondheim, Norway. They were carried out by fetal medicine specialists until 2005 and thereafter also by midwives specially trained in fetal embryology. Both groups were aware of the necessity of measuring the fetus in a neutral position, neither flexed nor hyperextended. Detailed data about the mother and her offspring, including the course of the pregnancy, delivery, birth and the neonatal period, have been prospectively collected and registered in the center's database since 1987. Selected employees have at all times managed the database and entered the data. The database now comprises a total of approximately 100 000 pregnancies with complete information on the ultrasound assessment of maternal and fetal clinical development. The majority of ultrasound examinations in our database are routine scans conducted at around 18 weeks' gestation. As part of the prospective data collection at the time of ultrasound examination and birth, information on smoking habits, maternal age, parity, number of fetuses, fetal anomalies, viability, sex of the child determined at birth, identity of the examiner, indication for the examination, delivery method and, where applicable, indications for induction of birth were also recorded.

This study of late first‐trimester and early second‐trimester dating includes data on all ultrasound examinations stored in the clinical database that had a valid CRL measurement in the range 5–84 mm and/or a valid BPD measurement in the range 5–28 mm, taken of a singleton fetus that resulted in a live birth without congenital malformation. The scans were part of the routine fetal examination with no particular medical indication, with a few exceptions that were excluded from our analysis, as detailed below. When extracted from the database, the analysis file was anonymized; all dates, location information, detailed diagnoses and other personal information were removed. This study was approved by the Regional Committees for Medical and Health Research Ethics (#2021/268077).

We extracted from the database 12 676 ultrasound examinations of 11 421 singleton fetuses. We excluded 360 pregnancies (395 examinations) that underwent induction of labor owing to maternal diabetes, growth restriction or a similar indication. Furthermore, 20 pregnancies (21 examinations) were excluded because the scan was performed for an indication possibly related to abnormal fetal growth. After all exclusions, our population comprised 11 041 pregnancies with 12 260 examinations. Among these, 7552 examinations had both CRL and BPD measurements in the required ranges, 4099 had only the CRL measurement in the required range and 609 had only the BPD measurement in the required range. Thus, there were a total of 11 651 CRL measurements in the range 5–84 mm and 8161 BPD measurements in the range 5–28 mm.

### Ultrasound examination

Ultrasound scans were performed using Hitachi EUB‐410, EUB‐415, EUB‐6000 and EUB‐6500 (Hitachi, Tokyo, Japan), Vingmed System Five (GE Vingmed Ultrasound, Horten, Norway) and LOGIQ 500 (GE Healthcare, Milwaukee, WI, USA) instruments with 5‐MHz curvilinear transducers.

CRL was measured transabdominally as part of the first‐trimester scan. The measurement was performed following standard criteria, with the fetus oriented horizontally on the screen so that the measurement line between crown and rump was about 90° to the ultrasound beam. The fetus was measured on a magnified image in a neutral position (i.e. neither flexed nor hyperextended). Calipers were placed at the end points of the crown and rump, which were visualized clearly.

In the first trimester, BPD was measured in the largest symmetrical axial view of the fetal head by placing calipers outer‐to‐outer, perpendicular to the midline falx at the level of the thalami. Techniques for measuring BPD in the range 5–20 mm have been described in detail previously^7^.

### Statistical analysis

#### Term prediction using local linear quantile regression for censored data

Traditional approaches to pregnancy dating on ultrasound, such as that recommended by Altman and Chitty^8^, start by collecting a number of ultrasound measurements spread evenly over the gestational‐age range for which prediction is to be performed. With gestational age computed from the last menstrual period (LMP), a prediction model is then constructed as a non‐linear regression of LMP‐based gestational age derived from the ultrasound measurement, for instance BPD. While well established, there are drawbacks to this approach, potentially causing a systematic bias in predictions when applied to population data^9^, ^10^. This issue is not easily resolved by simply predicting LMP‐based gestational age from ultrasound measurements in population data. The source of the problem is that ultrasound examinations in a population database are usually heavily selected towards the time of the examination, i.e. around week 12. Since mothers are typically invited to the examination based on their preliminary LMP‐based gestational‐age estimate, the selection is based precisely on the very variable that the model is trying to predict, again causing a bias in the regression.

To avoid these issues, we employed a population‐based approach that derives unbiased prediction models for the day of delivery, rather than LMP‐based gestational age, based on the first‐trimester ultrasound measurements of CRL and BPD. Following the strategy outlined by Gjessing *et al*.^11^, we first computed the remaining time of pregnancy as the number of days from the day of ultrasound examination until the day of birth. Then, we estimated the median remaining time of pregnancy as a non‐linear regression function of CRL and BPD. Models were developed separately for CRL and for BPD.

Traditional LMP‐focused models provide a potentially biased ultrasound estimate of LMP‐based gestational age, while our prediction models provide the EDD. While it is rarely documented, implementation of the traditional model will compute the EDD by subtracting predicted gestational age (A) from the assumed total average length of gestation (L). That is, the remaining time of pregnancy (R) is estimated as R = L–A. In our setting, R is estimated directly from the model as the median time from ultrasound examination to birth. Consequently, we compute predicted gestational age from the inverted formula, A = L–R. The assumed value of L is often not estimated as part of the prediction model; it is often chosen somewhat arbitrarily by programmers implementing the model. The value of 280 days (40 completed weeks) is frequently seen as the ‘official’ number, and 281 days also appears to be a common choice. In our data, we find the median estimate of L = 283 days. This is close to earlier findings^12^, ^13^, ^14^. Consequently, in our model, we derive estimated gestational age as A = 283–R.

The population‐based strategy uses the actual day of birth as its prediction target, thus avoiding the uncertainties and sampling biases associated with LMP. However, over recent decades, there has been a considerable increase in the proportion of births being induced, whether electively, for post‐term indications or for other indications. Clearly, EDD should refer to spontaneous births; non‐spontaneous onset of labor will shift the remaining time of pregnancy to lower values, potentially creating bias if not accounted for. Simply discarding non‐spontaneous births from the data might appear to solve this. However, in addition to being wasteful, it has the potential to cause bias. As an example, inductions for post‐term pregnancy happen precisely because these pregnancies have a very long duration; removing them from the analysis would thus systematically remove pregnancies that are known to be long, and the resulting median time to birth will be underestimated in the remaining data. This issue is well known in the context of TTE analysis, also known as event history analysis or survival analysis^15^. In our setting, non‐spontaneous births are seen as right‐censored; spontaneous births are events, and the timescale of a TTE analysis starts running from the day of the ultrasound examination. The idea behind this strategy is that the probability of having a spontaneous birth on any given day of pregnancy is calculated from the currently ongoing pregnancies, i.e. those not yet having experienced spontaneous birth or induction. Going back at least to 1970, similar strategies have been employed to study, for instance, the total duration of pregnancy^12^, ^16^, fetal death^17^ and factors that impact on gestational age and the risk of preterm birth^18^. The underlying assumption is that pregnancies with non‐spontaneous onset of birth would, had they not been induced, have proceeded following the same birth distribution as spontaneous births.

Statistical analysis was performed using R statistical software (R Foundation, Vienna, Austria)^19^. To estimate the value of the total median length of gestation (L), we applied a Kaplan–Meier estimate (*survfit* function in R) to the time from LMP to the time of birth, using only pregnancies for which the LMP was registered as both ‘certain’ and ‘regular’. Non‐spontaneous births were treated as censored. To develop the term prediction models, i.e. computing median remaining time from CRL or BPD values, we used a quantile regression model that allows for censored data, as implemented in the *crq* regression function from the R *quantreg* library^20^. However, since the *crq* regression model is primarily aimed at linear‐censored quantile regressions, we extended it to a local linear quantile regression^11^, ^21^, still allowing for censoring. We henceforth refer to this approach as the LLQRC approach. Technical details of the modeling are provided in Appendices S1–S7.

#### Evaluating impact of using time‐to‐event analysis

Our model‐fitting strategy treats non‐spontaneous births as censored by computing the median remaining time using a quantile regression allowing for censored observations. We investigated the possible bias caused by using simpler strategies to handle non‐spontaneous births. These strategies were: (1) computing the median from all births, not distinguishing between spontaneous and non‐spontaneous births; and (2) computing the median only from spontaneous births, removing non‐spontaneous births. The resulting median biases relative to the TTE analysis strategy were then plotted across the ranges of CRL and BPD values.

#### Comparing predictive precision of CRL and BPD

To compare the predictive precision of CRL and BPD, we used the subset of 7552 examinations in which both CRL and BPD were measured on the same day of pregnancy in the same fetus. As is customary for prediction models, we compared residual distributions to evaluate predictive quality. Residuals were computed as the observed remaining time minus the median remaining time as determined from the model. That is, residuals are the actual time of birth relative to the predicted term. We then estimated an overall cumulative residual birth distribution (F(t)) by computing F(t) = 1–S(t), where S(t) is the Kaplan–Meier estimate^15^ of the ‘survival function’ and t is the residual remaining time. Non‐spontaneous births were again treated as censored observations. The advantage of considering the estimated cumulative residual distribution rather than the residual distribution itself is the possibility of accounting for censoring. It should be noted that, when accounting for censoring with the Kaplan–Meier approach, the resulting estimate of F(t) represents the birth distribution around term as it would have been observed in a situation in which no births were induced. To obtain the corresponding estimate of the residual distribution itself, we used the R software function *smooth.spline* to calculate the derivative of F(t).

Since CRL and BPD were measured on the same day of pregnancy, we could compare the two predictions directly on the same data. It should be noted that if R_CRL_ and R_BPD_ are remaining times of pregnancy estimated from CRL and BPD, respectively, then A_BPD_–A_CRL_ = (L–R_BPD_)–(L–R_CRL_) = R_CRL_–R_BPD_; the difference in predicted gestational age is the same as the difference in predicted remaining time, except with opposite signs. In addition, the difference is independent of whether births were induced or not. Thus, it is sufficient to quantify the gestational‐age difference to compare the methods. We counted the number of pregnancies with zero difference, 1 day difference and so forth, and plotted the corresponding histogram.

In addition, we investigated whether a higher total prediction quality could be achieved by using predictions based on a combination of CRL and BPD measurements. This was done by checking if median prediction residuals for the CRL prediction changed over categories of BPD, and *vice versa*. In a similar fashion, since the registry period spans 30 years, we checked if there were any systematic trends in prediction residuals over time. Further information is provided in Appendices S5 and S6.

## RESULTS

There were 6187 pregnancies for which CRL and/or BPD were available in the prescribed ranges and for which the mother also reported the LMP as ‘certain’ and ‘regular’. When accounting for inductions as censoring, the median total length of gestation in this group was 282.8 (95% CI, 282.5–283.1) days. Our model for calculating gestational‐age predictions uses the rounded value of 283 days.

**Figure 1:**
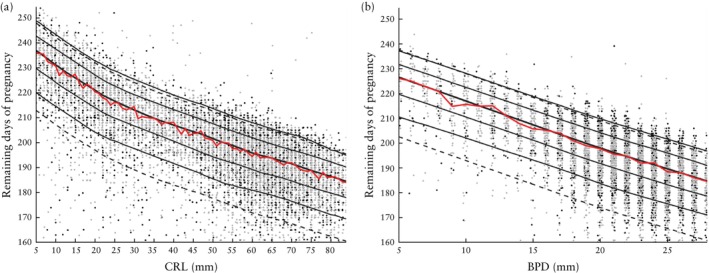
(a) Remaining days of pregnancy plotted against crown–rump length (CRL) measured in 11 651 ultrasound examinations with CRL in the range 5–84 mm. (b) Remaining days of pregnancy plotted against biparietal diameter (BPD) measured in 8161 ultrasound examinations with BPD in the range 5–28 mm. , Spontaneous births; , induced births. Solid black lines, from bottom up, indicate 10^th^, 25^th^, 50^th^ (median, thick line), 75^th^ and 90^th^ percentiles of remaining days, estimated by local linear quantile regression for censored data model. Dashed lines, from bottom up, indicate limit of preterm birth, defined as median remaining time minus 24 days, and limit of post‐term birth, defined as median remaining time plus 11 days. Red line is median remaining time calculated from Kaplan–Meier estimates separately for each mm of CRL or BPD.

The LLQRC method produced stable and smooth estimates of median remaining time over the full prediction range for both CRL and BPD. Figure 1a shows the remaining days of pregnancy plotted against CRL measured at the examination, over the range 5–84 mm. Added to the plot are lines showing a selection of percentile curves also estimated using the LLQRC method. Across the prediction range 35–84 mm, the curves are almost linear. Furthermore, the percentile curves are nearly parallel, implying that the prediction uncertainty is almost constant across the range of CRL values. In addition to the percentile curves, the curves defining preterm and post‐term births are indicated; these were computed by subtracting 24 days or adding 11 days, respectively, to the median curve, thus corresponding to a gestational age of 259 and 294 days, respectively. The predicted number of remaining days and the corresponding predicted gestational age can be found in Appendix S1.

**Figure 2:**
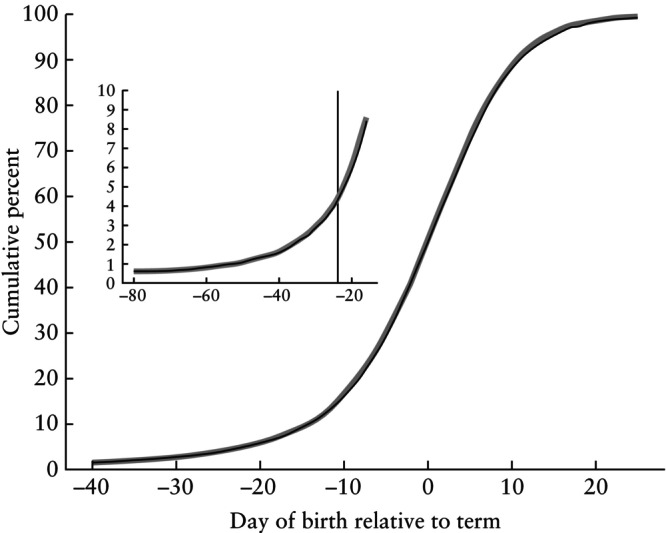
Cumulative birth distribution curves based on 7552 examinations in which both crown–rump length (CRL) (range, 5–84 mm) and biparietal diameter (BPD) (range, 5–28 mm) were measured on the same day and used for pregnancy dating. Cumulative distributions were computed separately for CRL and BPD using a time‐to‐event approach, treating non‐spontaneous births as censored. Inset shows an enlarged plot of preterm region (–80 days to –16 days). Thick gray line is CRL and black line is BPD. Vertical line at –24 days marks first day of term period, corresponding to 259 days on gestational‐age scale.

**Figure 3:**
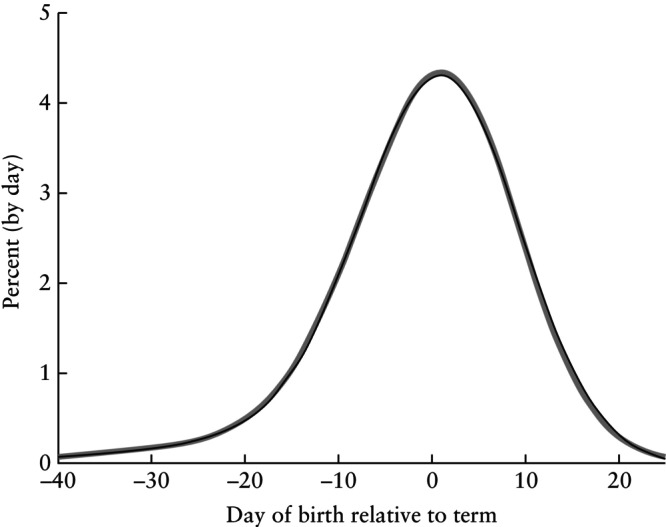
Estimated birth distribution curves based on 7552 examinations in which both crown–rump length (CRL) (range, 5–84 mm) and biparietal diameter (BPD) (range, 5–28 mm) were measured on the same day and used for pregnancy dating. Distribution curves were calculated as the derivative (slope) of the cumulative curves shown in Figure 2. Thick gray line is CRL and black line is BPD.

Figure 1b shows the corresponding results when predicting the remaining days of pregnancy from BPD measured at the examination, over the range 5–28 mm. Across the entire BPD prediction range, the curves are nearly linear and parallel, thus again demonstrating that prediction uncertainty is relatively stable across BPD values. The predicted number of remaining days and the corresponding predicted gestational age can be found in Appendix S1. Note that a comparison of Figures 1a and 1b might give the impression that BPD is notably more precise than CRL since there are, for instance, more births falling below the line demarcating the preterm region in Figure 1a. However, the larger number of preterm births is simply due to having more CRL measurements in total; the percentages of preterm births by CRL and BPD are nearly identical, as seen in Figures 2 and 3.

To compare the precision of the CRL and BPD predictions, we compared the residual distributions when estimating time of birth. Values of F(t) (computed as F(t) = 1–S(t)), are shown in Figure 2. Figure 3 shows the corresponding frequency distribution curves, calculated from the estimated cumulative curves. The residual distributions are almost identical for CRL and BPD.

**Figure 4:**
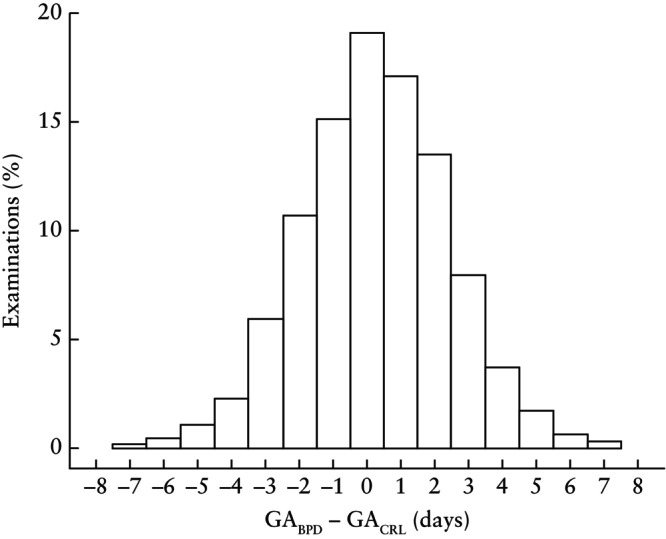
Difference (in days) between ultrasound‐predicted gestational age (GA) using biparietal diameter (GA_BPD_) (range, 5–28 mm) and GA using crown–rump length (GA_CRL_) (range, 5–84 mm), based on 7552 examinations in which both BPD and CRL were measured on the same day. Outliers are not plotted.

By computing individual differences between CRL‐ and BPD‐based predictions, where CRL and BPD were measured on the same day, we could assess by how many days the gestational age and term predictions of any given fetus would differ between the two methods. Figure 4 provides a histogram of the difference between CRL‐ and BPD‐based predictions of remaining time and gestational age. The symmetry of the histogram around zero shows that neither of the methods produces estimates that are systematically larger or smaller than the other, and the differences are typically small. In 50.8% of examinations, the difference was 1 day or less; 23.8% of examinations had a difference of 2 days, 13.9% had a difference of 3 days and 6.5% had a difference of 4 days. Beyond that, only 5.0% of all examinations had a difference of 5 days or more.

Appendix S2 shows the time trend in the percentage of non‐spontaneous births, as well as the distribution of inductions according to gestational age, and whether the indication for induction was post‐term pregnancy or otherwise.

Appendix S3 demonstrates the biases that would result from not properly accounting for induced births in the estimation method. The median bias computed using the TTE method is shown. In addition, we show the biases resulting from two other options: computing the median bias from all data, disregarding whether birth was induced or not, and computing the median bias from only spontaneous births. It is clear that the simple approaches of either excluding all non‐spontaneous births or including all but disregarding the fact that they were induced will typically result in a bias of –2 days, indicating that these approaches would make the median remaining time appear 2 days shorter than when inductions are properly accounted for.

To illustrate the potential bias of the approach predicting LMP‐based gestational age using ultrasound in a population setting, the results of computing median LMP‐based gestational age for each mm of CRL and BPD are shown in Appendix S7, compared with the gestational age estimated from the LLQRC model. The figures show that the median LMP‐based gestational age is biased towards a gestational age of 12 + 3 weeks, since mothers are actually invited to the week‐12 examination based on their LMP‐based gestational age.

We found no indication that combining CRL and BPD in a single prediction model would improve the total prediction quality. There was also no evidence of any systematic trend in prediction bias over the birth year period from 1987 to 2017. Further details are provided in Appendices S5 and S6.

## DISCUSSION

While CRL is clinically well established as a parameter for first‐trimester ultrasound dating, BPD has to a large extent been used in the second trimester. In the present study, we focused on evaluating BPD as a feasible alternative to CRL for week‐12 dating. The quality of BPD‐ and CRL‐based dating around week 12 was compared directly, using the same population and the same statistical approach, to derive prediction curves.

As shown in our previous studies^9^, ^10^, the traditional strategy of predicting LMP‐based gestational age leads to biased predictions in a population setting^22^. Our approach circumvents this problem by first predicting the day of delivery, followed by calculating the corresponding gestational age. Consequently, our approach is valid in a population setting in which it is applied routinely in pregnancy care^11^, ^23^.

Any method attempting to predict the day of delivery is faced with a challenge: a substantial number of births are induced before the time at which they would have taken place spontaneously. To overcome this problem, we used a TTE analysis approach, in which inductions were treated as censored. This was done both in the development of the prediction model itself, as well as in the assessment of the final residual distributions.

Our findings clearly suggest that, as a measure for pregnancy dating around week 12, BPD and CRL provide the same precision. While Figure 4 shows that one might expect a few days' difference between predictions from BPD and CRL, it does not provide evidence that one measure is better than the other. The residual distributions, as shown in Figures 2 and 3, are practically identical. Based on the statistical evaluation, BPD may thus be a reliable replacement for CRL.

In the study by Chalouhi *et al*.^24^, a limited dataset of pregnancies conceived by assisted reproductive technology was used to develop gestational‐age prediction formulae. They tested the formulae on term prediction in a population dataset. While their prediction model was not population‐based, their conclusion was similar to ours: CRL and BPD had comparable precision, whereas head circumference and abdominal diameter showed lower precision. Another study followed our approach of predicting term directly from ultrasound measurements and, while not looking at the prediction differences at an individual level, it concluded that CRL and BPD had comparable precision at the population level^23^. A study comparing the reproducibility of CRL and BPD in the first trimester noted that the reproducibility of CRL declines with increasing gestational age, apparently in line with the increasing difficulty of finding a neutral position for the fetus when measuring the CRL^6^. Other studies have provided similar results^25^, ^26^.

From a clinical and practical point of view, there are good reasons to recommend BPD over CRL for late‐first‐trimester dating. First, the contour of the bony fetal skull is depicted clearly in the image, and the reference points – the falx cerebri and thalami – are easily recognized. Second, in contrast to CRL, a BPD measurement is not affected by an extended or a flexed fetus, and thus saves waiting time. Third, BPD remains important up until birth. In a clinical follow‐up of fetal growth, curves to assess fetal size at any gestational age will typically include BPD as a key parameter^27^. It is an advantage to use the same parameter for pregnancy dating as for size assessment later in gestation, because it improves the ability to precisely detect deviations from normal growth. Fourth, the ease of use of BPD implies that a routine examination at week 12 or 13 will provide precise dating, as well as a better opportunity for a full anatomical review, and will thus improve the chance of detecting fetal anomalies.

A practical aspect related to the use of BPD relative to CRL is that there is a difference in scale. As seen in Appendix S1, the gestational‐age range from 12 + 0 to 12 + 6 weeks corresponds to a change in CRL from 55 to 67 mm, that is, a span of 12 mm. The corresponding change for BPD is from 19.5 to 23.0 mm, that is, a span of 3.5 mm. In relative terms, the CRL increase is 22%, whereas the BPD increase is 18%. In absolute terms, however, the CRL span is more than three times that of BPD. Therefore, it is important to make sure that BPD is measured and recorded at a finer scale than 1 mm, for example in steps of 0.5 mm.

The data utilized in the present study, derived from the National Center for Fetal Medicine in Trondheim, Norway, were produced by uniformly trained and highly experienced ultrasound operators. While BPD is a parameter that is technically easier and faster to measure, CRL requires more experience, training and dedicated time to measure. It is thus likely that less experienced operators are at risk of introducing biases (not seen in this study) when measuring CRL, and that they will benefit from the relative simplicity of measuring BPD.

In conclusion, our study finds that BPD is as precise as CRL when used for first‐trimester dating. While CRL has been the preferred choice for first‐trimester ultrasound dating, our study highlights the advantages of using BPD. From a clinical and practical point of view, there are many reasons to promote the use of BPD for first‐trimester dating. It is a tried‐and‐tested parameter for second‐ and third‐trimester assessment, and there is an obvious advantage to using the same parameter throughout pregnancy. Furthermore, BPD is technically less challenging and less time‐consuming to measure compared with CRL. It is therefore timely to advance a more prominent role for BPD in late‐first‐trimester pregnancy dating.

## Supporting information


**Appendix S1** Prediction tables and simplified formulae
**Appendix S2** Distribution of inductions over time and gestational age
**Appendix S3** Evaluating impact of using time‐to‐event analysis
**Appendix S4** Statistical methods, further details
**Appendix S5** Checking predictive power of crown–rump length (CRL) in combination with biparietal diameter (BPD)
**Appendix S6** Checking for trends in data
**Appendix S7** Biases when using last menstrual period‐based gestational age at examination in model development

## Data Availability

The data are not publicly available due to privacy or ethical restrictions.
