# Supplementary material for: Biparietal diameter vs crown–rump length as standard parameter for late first‐trimester pregnancy dating
Source: Ultrasound Obstet Gynecol. 2024 Oct 24;64(6):739–45. doi: 10.1002/uog.29124 (PMC11609919; doi:10.1002/uog.29124)
Supplement: Supplementary file 1 — Appendix S1 Prediction tables and simplified formulae Appendix S2 Distribution of inductions over time and gestational age Appendix S3 Evaluating impact of using time‐to‐event analysis Appendix S4 Statistical methods, further details Appendix S5 Checking predictive power of crown–rump length (CRL) in combination with biparietal diameter (BPD) Appendix S6 Checking for trends in data Appendix S7 Biases when using last menstrual period‐based gestational age at examination in model development [file UOG-64-739-s001.pdf]

H. K. Gjessing, P. Grøttum, J. M. Dreier, S. H. Eik-Nes

Biparietal diameter vs. crown-rump length as standard parameter for late first trimester pregnancy dating

Online supporting information

Appendix S1

Prediction tables and simplified formulas

Tables S1 and S2 below contain predicted values of remaining days and gestational age for CRL and BPD, respectively. Predicted remaining days were first computed using the LLQRC model described in the main article and Appendix S4 below, and then GA was estimated as $GA = 283 - RE$. However, since the LLQRC method itself does not provide simple formulas to compute the prediction, we supply "simplified formulas" with which to compute the table values. The simplified formulas are derived by fitting a fractional polynomial equation directly to the *predicted* GA values deriving from the LLQRC method. The fractional polynomials are thus not used in the smoothing model itself; they only provide means to an easy calculation of the table values, which is useful, for instance, in ultrasound machine implementations. The values computed from the polynomials differ from the original LLQRC values by less than 0.4 days. When the polynomial formula has been used to compute GA, the estimated remaining time can be computed from $RE = 283 - GA$.

Supplementary Table S1

Predictions based on crown-rump length (CRL, **5–84 mm**).
n: number of scans used in estimation.
RE: estimated remaining days of pregnancy, and completed weeks/days
GA: estimated days of gestational age at ultrasound, and completed weeks/days.
Simplified formula for GA (RE is calculated from RE = 283 - GA):

$$GA = 84 - 108.11 \cdot CRL^{1/3} + 70.762 \cdot CRL^{1/2} - 2.3605 \cdot CRL + 0.0053775 \cdot CRL^2$$

CRL	n	RE	RE(w/d)	GA	GA(w/d)
5	102	237.30	33/6	45.70	6/4
6	92	236.09	33/5	46.91	6/5
7	82	234.85	33/4	48.15	6/6
8	82	233.61	33/3	49.39	7/0
9	101	232.40	33/1	50.60	7/2
10	131	231.21	33/0	51.79	7/3
11	104	230.06	32/6	52.94	7/4
12	122	228.93	32/5	54.07	7/5
13	99	227.84	32/4	55.16	7/6
14	105	226.79	32/3	56.21	8/0
15	108	225.76	32/2	57.24	8/1
16	85	224.76	32/1	58.24	8/2
17	117	223.80	32/0	59.20	8/3
18	108	222.86	31/6	60.14	8/4
19	119	221.94	31/5	61.06	8/5
20	162	221.06	31/4	61.94	8/6
21	118	220.19	31/3	62.81	9/0
22	142	219.35	31/2	63.65	9/1
23	126	218.53	31/2	64.47	9/1
24	133	217.74	31/1	65.26	9/2
25	113	216.96	31/0	66.04	9/3
26	91	216.20	30/6	66.80	9/4
27	88	215.45	30/5	67.55	9/5
28	104	214.73	30/5	68.27	9/5
29	80	214.02	30/4	68.98	9/6
30	89	213.32	30/3	69.68	10/0
31	69	212.64	30/3	70.36	10/0
32	92	211.97	30/2	71.03	10/1
33	88	211.31	30/1	71.69	10/2
34	85	210.67	30/1	72.33	10/2
35	77	210.03	30/0	72.97	10/3
36	78	209.41	29/6	73.59	10/4
37	73	208.79	29/6	74.21	10/4
38	78	208.19	29/5	74.81	10/5
39	72	207.60	29/5	75.40	10/5
40	99	207.01	29/4	75.99	10/6
41	77	206.43	29/3	76.57	11/0
42	86	205.86	29/3	77.14	11/0
43	85	205.29	29/2	77.71	11/1
44	89	204.74	29/2	78.26	11/1
45	110	204.18	29/1	78.82	11/2
46	106	203.64	29/1	79.36	11/2
47	136	203.09	29/0	79.91	11/3
48	142	202.56	29/0	80.44	11/3
49	121	202.03	28/6	80.97	11/4
50	155	201.50	28/5	81.50	11/5
51	156	200.98	28/5	82.02	11/5
52	180	200.45	28/4	82.55	11/6
53	200	199.94	28/4	83.06	11/6
54	222	199.42	28/3	83.58	12/0
55	200	198.91	28/3	84.09	12/0
56	240	198.40	28/2	84.60	12/1
57	221	197.90	28/2	85.10	12/1
58	268	197.39	28/1	85.61	12/2
59	237	196.89	28/1	86.11	12/2
60	331	196.39	28/0	86.61	12/3
61	242	195.89	28/0	87.11	12/3
62	308	195.39	27/6	87.61	12/4
63	304	194.89	27/6	88.11	12/4
64	307	194.39	27/5	88.61	12/5
65	291	193.89	27/5	89.11	12/5
66	310	193.39	27/4	89.61	12/6
67	253	192.90	27/4	90.10	12/6
68	246	192.40	27/3	90.60	13/0
69	234	191.90	27/3	91.10	13/0
70	252	191.40	27/2	91.60	13/1
71	202	190.90	27/2	92.10	13/1
72	226	190.40	27/1	92.60	13/2
73	185	189.90	27/1	93.10	13/2
74	169	189.39	27/0	93.61	13/3
75	163	188.89	27/0	94.11	13/3
76	152	188.38	26/6	94.62	13/4
77	150	187.88	26/6	95.12	13/4
78	113	187.37	26/5	95.63	13/5
79	123	186.85	26/5	96.15	13/5
80	138	186.34	26/4	96.66	13/6
81	77	185.83	26/4	97.17	13/6
82	76	185.31	26/3	97.69	14/0
83	77	184.79	26/3	98.21	14/0
84	77	184.26	26/2	98.74	14/1

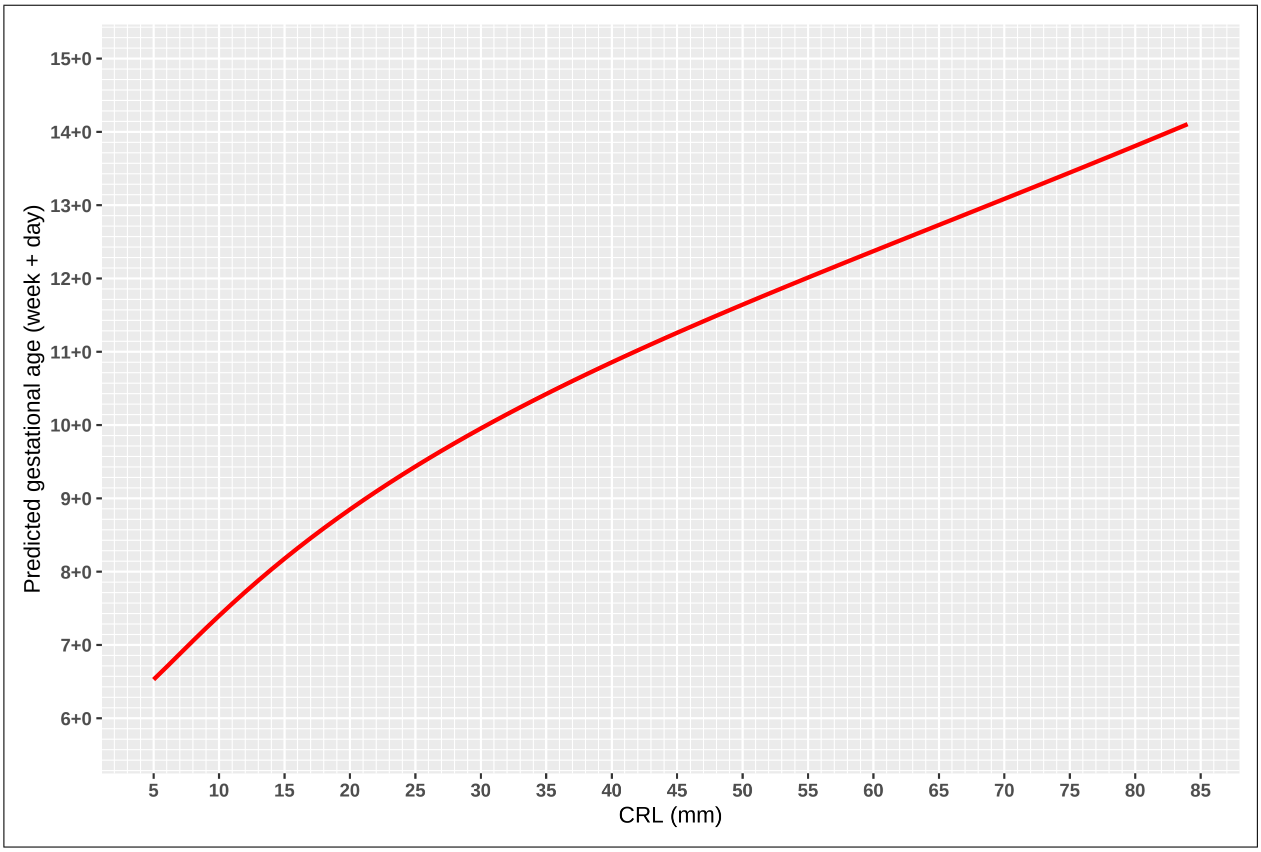

Supplementary Figure 1: *Predicted gestational age from CRL (crown-rump length). Based on 11 651 ultrasound examinations.*

Supplementary Table S2

Predictions based on biparietal diameter (BPD, **5–28 mm**).
n: number of scans used in estimation.
RE: estimated remaining days of pregnancy, and completed weeks/days.
GA: estimated days of gestational age at ultrasound, and completed weeks/days.
Simplified formula for GA (RE is calculated from RE = 283 - GA):

$$GA = 46.25 + 2.0575 \cdot BPD - 0.00692 \cdot BPD^2$$

BPD	n	RE	RE(w/d)	GA	GA(w/d)
5.0	45	226.64	32/3	56.36	8/0
5.5	0	225.64	32/2	57.36	8/1
6.0	49	224.65	32/1	58.35	8/2
6.5	0	223.67	32/0	59.33	8/3
7.0	65	222.69	31/6	60.31	8/4
7.5	0	221.71	31/5	61.29	8/5
8.0	65	220.73	31/4	62.27	8/6
8.5	0	219.76	31/3	63.24	9/0
9.0	71	218.79	31/2	64.21	9/1
9.5	0	217.83	31/1	65.17	9/2
10.0	93	216.87	31/0	66.13	9/3
10.5	0	215.91	30/6	67.09	9/4
11.0	85	214.95	30/5	68.05	9/5
11.5	0	214.00	30/4	69.00	9/6
12.0	113	213.06	30/3	69.94	10/0
12.5	0	212.11	30/2	70.89	10/1
13.0	105	211.17	30/1	71.83	10/2
13.5	0	210.23	30/0	72.77	10/3
14.0	133	209.30	29/6	73.70	10/4
14.5	0	208.37	29/5	74.63	10/5
15.0	163	207.44	29/4	75.56	10/6
15.5	0	206.52	29/4	76.48	10/6
16.0	187	205.60	29/3	77.40	11/0
16.5	0	204.69	29/2	78.31	11/1
17.0	252	203.77	29/1	79.23	11/2
17.5	0	202.86	29/0	80.14	11/3
18.0	364	201.96	28/6	81.04	11/4
18.5	0	201.05	28/5	81.95	11/5
19.0	502	200.16	28/4	82.84	11/6
19.5	0	199.26	28/3	83.74	12/0
20.0	634	198.37	28/2	84.63	12/1
20.5	0	197.48	28/1	85.52	12/2
21.0	729	196.59	28/1	86.41	12/2
21.5	0	195.71	28/0	87.29	12/3
22.0	861	194.83	27/6	88.17	12/4
22.5	0	193.96	27/5	89.04	12/5
23.0	879	193.09	27/4	89.91	12/6
23.5	0	192.22	27/3	90.78	13/0
24.0	756	191.36	27/2	91.64	13/1
24.5	0	190.49	27/1	92.51	13/2
25.0	665	189.64	27/1	93.36	13/2
25.5	0	188.78	27/0	94.22	13/3
26.0	574	187.93	26/6	95.07	13/4
26.5	0	187.09	26/5	95.91	13/5
27.0	416	186.24	26/4	96.76	13/6
27.5	0	185.40	26/3	97.60	14/0
28.0	355	184.57	26/3	98.43	14/0

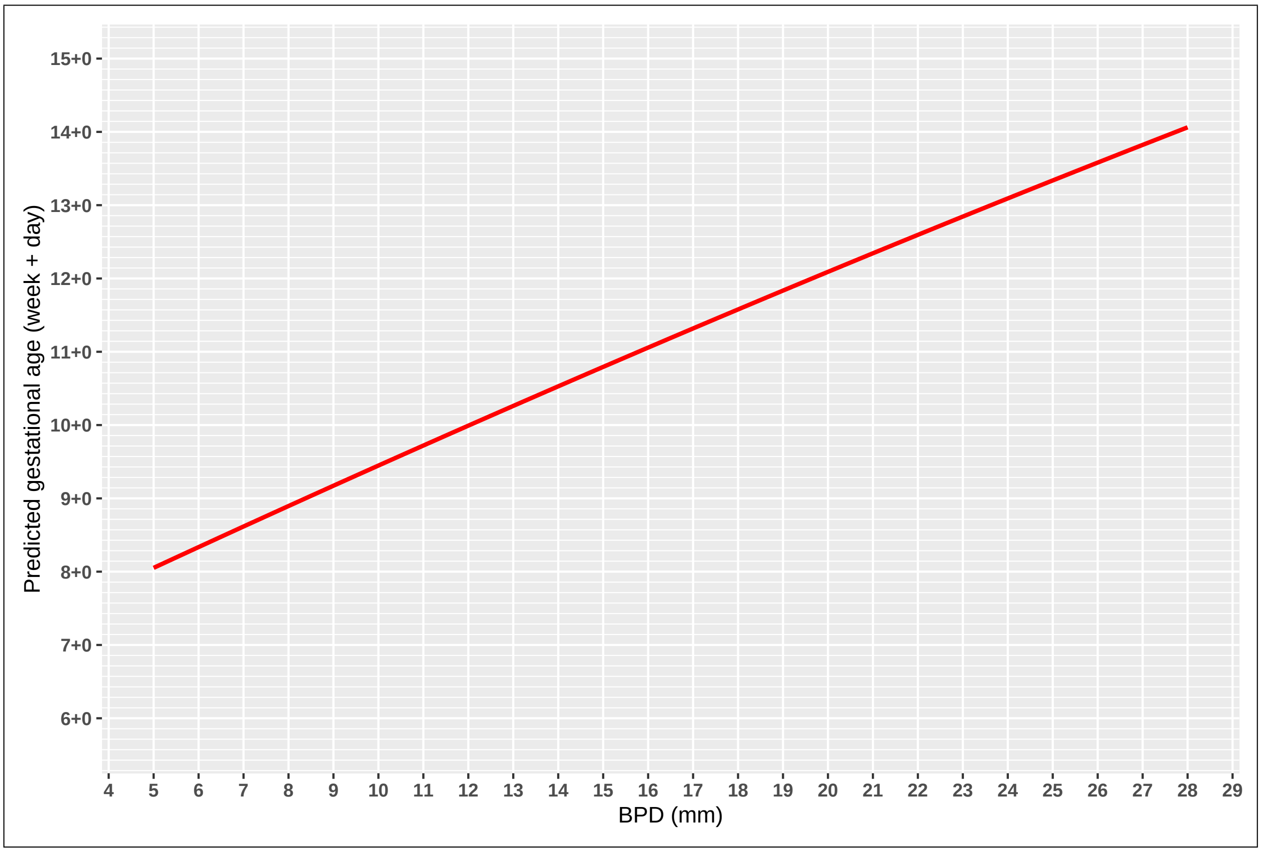

Supplementary Figure 2: *Predicted gestational age from BPD (biparietal diameter). Based on 8 161 ultrasound examinations.*

Appendix S2

Distribution of inductions over time and gestational age

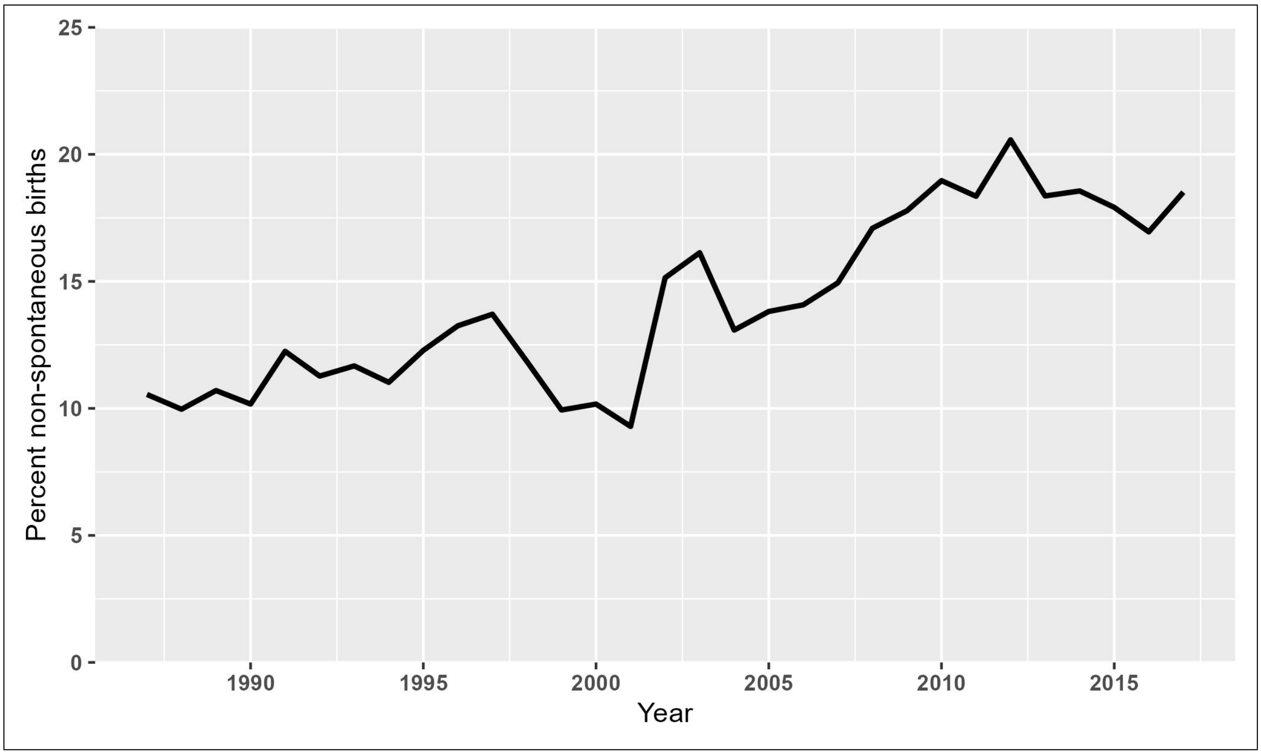

Supplementary Figure 3: *Time trend in the percentage of non-spontaneous births. Based on 81 116 pregnancies, 1987–2017.*

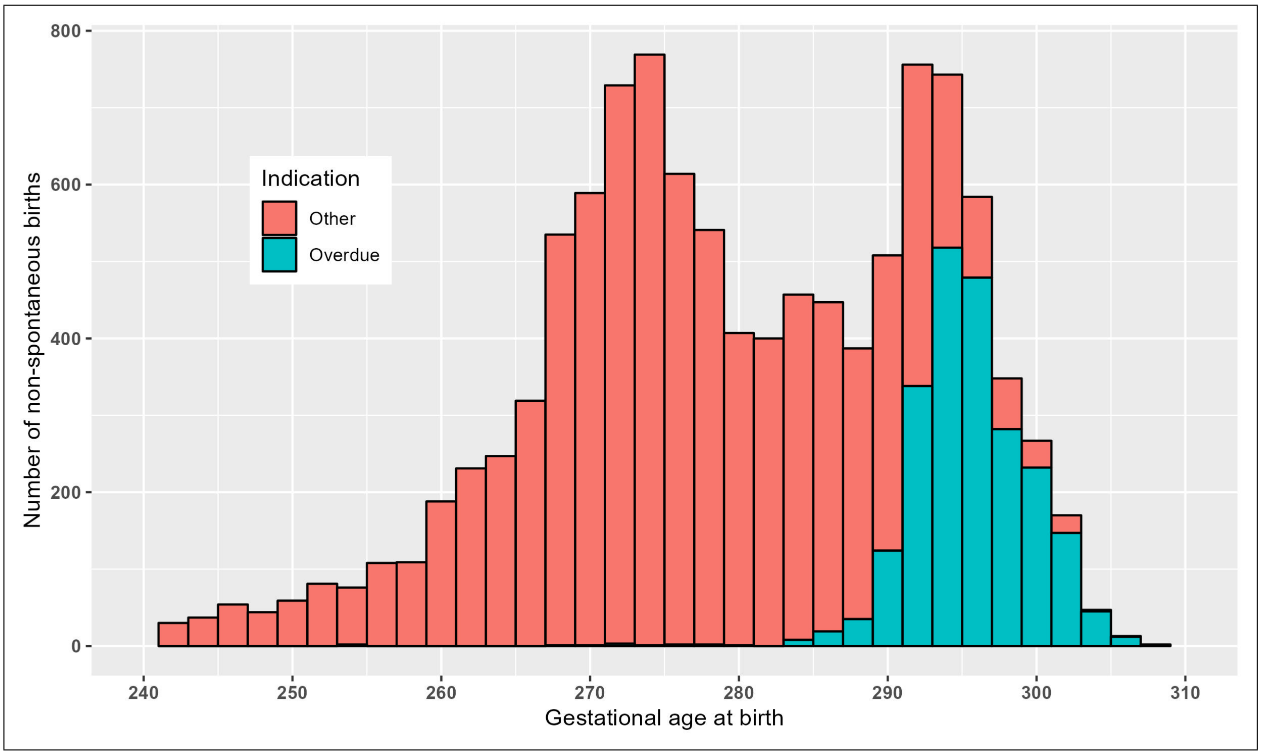

Supplementary Figure 4: *Stacked histogram of the distribution of the total number of non-spontaneous births by gestational age, split according to whether the induction indication was "overdue" or something else. Based on 81 116 pregnancies, 1987–2017.*

Appendix S3

Evaluating the impact of using time-to-event analyses

As described in the main text, a clear, systematic bias appears if inductions are not properly accounted for as censoring in a time-to-event analysis. The two panels in Supplementary Figure 5 demonstrate this issue.

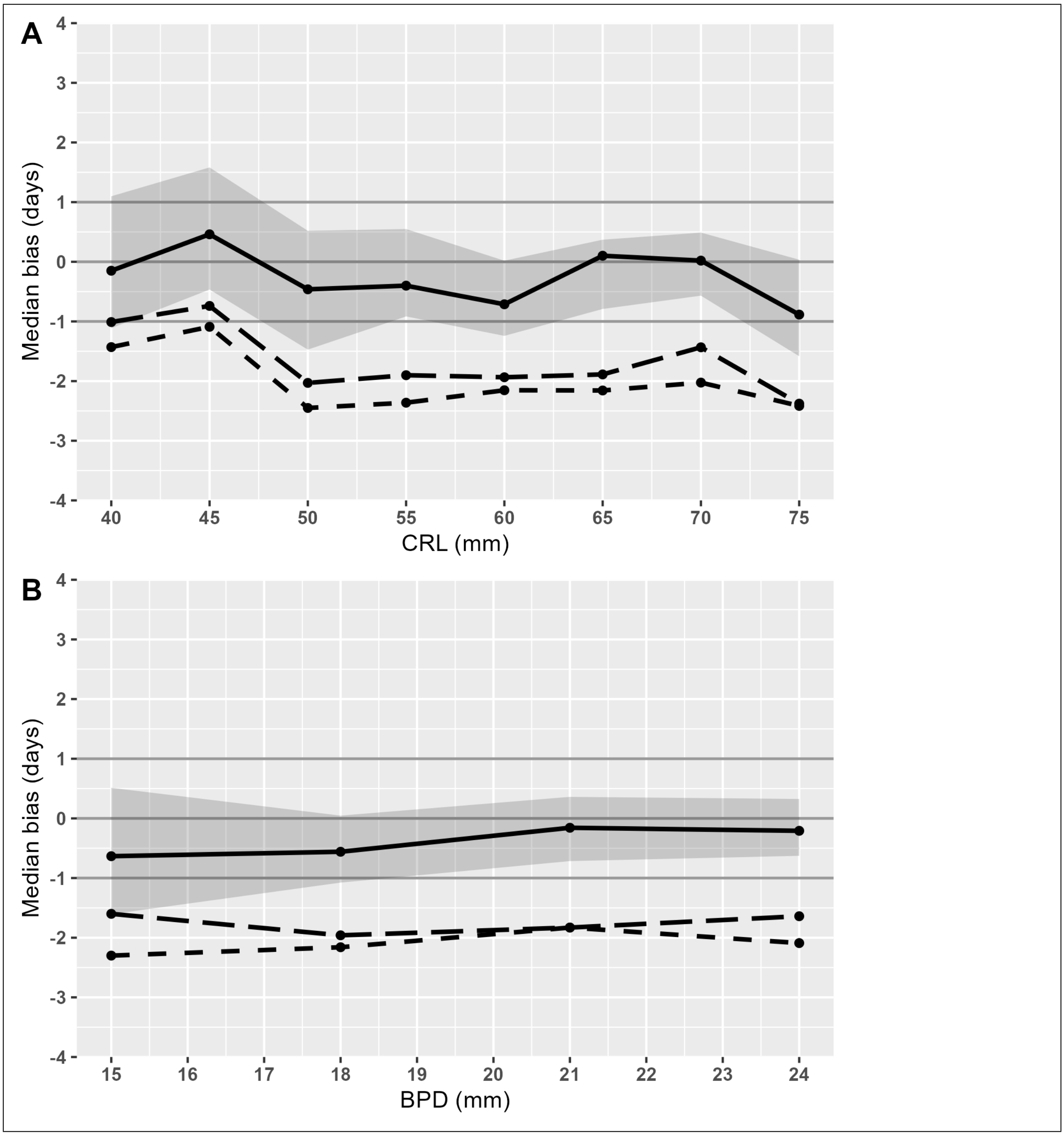

Supplementary Figure 5: *Median bias across the crown-rump length (CRL) and biparietal diameter (BPD) ranges for three different approaches to handling non-spontaneous onset of birth. Panel A: CRL prediction, with bias computed in groups of 5 mm CRL. Panel B: BPD predictions, with bias computed in groups of 3 mm BPD. Solid line: median prediction residuals (bias) computed using a Kaplan-Meier median estimate in each group, treating non-spontaneous births as censored. Grey shaded background shows 95% confidence intervals. Long dashed line: computing the median bias only from spontaneous births, removing non-spontaneous births. Short dashed line: computing the median bias from all births, not distinguishing between spontaneous and non-spontaneous birth.*

Appendix S4

Statistical methods, further details

The essential quantity to be estimated is the median remaining time RE from ultrasound examination until birth, as a smoothed function of CRL or BPD. The method should properly account for induced births as "censored" observations in a time-to-event type analysis. In the following, we describe the details of our analysis for CRL; the same method was applied to BPD.

The LLQRC regression models The *crq* function from the *quantreg* package (Koenker, 2023) in the R statistical software (R Core Team, 2022) performs linear quantile regressions that allow for censored observations. In particular, it can estimate the median as the 50% quantile, predicted as a linear function of CRL. However, since median remaining time RE is not a linear function in CRL over the entire CRL prediction range, a more flexible extension of the *crq* is needed. Based on the *crq* function, we implemented a local linear regression approach that performs a series of *crq*s, one for each value of CRL. In the following, we refer to this approach as the LLQRC method, as an extension of our previously employed method LLQR (Gjessing et al., 2007; Yu and Jones, 1998). The LLQRC method estimates median RE as a non-linear smooth function of CRL, incorporating the RE of non-spontaneous births as censored observations at the time of birth. For a single chosen CRL value, CRL_0, the LLQRC is performed as a weighted linear quantile regression with censoring around CRL_0, using weights of the form $\psi(CRL - CRL_0)$, where $\psi(x)$ is a normal distribution with mean zero and variable width (standard deviation, SD). The weights $\psi(CRL - CRL_0)$ are thus largest for scans with CRL value equal to CRL_0, and decreases towards zero when CRL increases far enough above (or decreases far enough below) CRL_0. The estimated median RE value was then computed from the regression as the predicted value when $CRL = CRL_0$. This regression procedure was repeated for all values of CRL_0 in the prediction range, allowing the level of smoothing to increase at the lower end of the CRL range, where there are fewer data points (Yu and Jones, 1998).
In addition to the median, we estimated a selection of other quantiles as functions of CRL. This provides an impression of how the spread of the RE distribution changes over the range of CRL. The estimates were computed by the same approach as for the median, but all quantiles were computed separately, with somewhat higher bandwidths for the more extreme quantiles. To regress remaining time on BPD, we followed the same procedure as described above for the CRL, both for the median prediction and for the other quantiles.

Evaluating model fit and prediction quality A familiar problem of flexible regression models is the risk of *overfitting* the model to the data (Hastie et al., 2001). If a regression model inherently has a high degree of freedom, and such a model is fitted to a relatively small dataset, there is the possibility of the model being fitted to follow the random variation in the data, rather than the correct underlying structure, which may be obscured by noise. This is particularly likely if the number of model parameters far exceeds the number of observations in the data file, commonly know as $p >> n$ problems. In such cases it is often recommended to use procedures such as crossvalidation to get a more correct picture of the residual distribution. Or more transparently, to use two separate data files for model development (training dataset) and prediction quality check (test dataset), where the two data files can be generated from a random split of the original data file.
In our setting, however, albeit the model is relatively complex, the resulting prediction curves are very smooth and depend only on a single predictor, either CRL or BPD. In addition, the dataset is quite large relative to the smoothness of the curves. The practical consequence of this is that splitting the data file into a training set and a test set is not needed; the resulting residual curves on the test set would be nearly identical to those that we find in the total dataset, shown in Figures 2 and 3 in the main article. Accordingly, we have used the entire data file both for fitting the model and for evaluation the residual distributions.

Appendix S5

Checking the predictive power of CRL in combination with BPD

The two prediction models were developed independently of one another, using either all available CRL measurements or all available BPD measurements. While most ultrasound prediction models for gestational age rely on a single variable at a time, it is natural to ask whether CRL measurements provide additional predictive power above and beyond what is provided by BPD alone, or similarly, whether BPD might add predictive power on top of what CRL alone provides.
To investigate this in our data, we used the subset of 7 552 examinations where both CRL and BPD were measured at the same time. First, we computed residuals from the CRL prediction, i.e. the number of remaining days of pregnancy minus the predicted number of remaining days. Then, we checked if the systematic prediction bias, i.e. median residual, changed across categories of BPD. Similarly, we computed residuals from the BPD prediction, and checked if prediction bias changed across categories of CRL.
The results were as follows:

```
Median CRL prediction bias in groups of BPD (with 95% CI)
BPD group  Median bias         95% CI
   [5,12]        -0.46  (-0.99, 0.71)
  (12,21]         0.11  (-0.32, 0.60)
  (21,28]        -0.40  (-0.86, 0.09)
```

```
Median BPD prediction bias in groups of CRL (with 95% CI)
CRL group  Median bias         95% CI
   [5,31]         1.00   (0.12, 1.86)
  (31,60]        -0.16  (-0.60, 0.40)
  (60,84]        -0.08  (-0.37, 0.27)
```

No median prediction bias exceeds 1 day, and with the exception of the BPD prediction bias when CRL is in the range 5–31 mm, they are all below 0.5 days. Thus, there is no indication that a combination of CRL and BPD in predictions would provide improved precision. This conclusion is in line with observations made previously (Taipale and Hiilesmaa, 2001).

Appendix S6

Checking for trends in data

The data cover a time period of 30 years, from 1987 to 2017. While a rigorous standard of training and measurement has been central at the National Center for Fetal Medicine, it is important to check for possible systematic changes in data and measurements over time. We followed the same approach as in Appendix S5 above and computed residuals from the CRL predictions, this time using all available data on CRL alone for the CRL predictions. We then checked if the prediction bias (median residual) changed across categories of birth year, and performed a similar check for the BPD predictions.
The results were as follows:

```
Median CRL prediction bias in groups of birth year (with 95% CI)
Birth year group  Median bias         95% CI
     [1987,2000]         0.11   (-0.38, 0.64)
     (2000,2010]         0.21   (0.06,  0.60)
     (2010,2017]        -0.59  (-0.91, -0.39)
```

```
Median BPD prediction bias in groups of birth year (with 95% CI)
Birth year group  Median bias         95% CI
     [1987,2000]       -0.149 (-1.294, 0.661)
     (2000,2010]        0.465  (0.148, 0.879)
     (2010,2017]       -0.383 (-0.844, 0.007)
```

The biases remain within 0.6 days, which we consider sufficiently small to be unimportant.

Appendix S7

Biases when using last menstrual period gestational age at examination in model development

Conventional model development has been based on predicting last menstrual period (LMP) gestational age at examination from the ultrasound measurement, and then computing the implied term prediction from the estimated gestational age. This has typically been performed by obtaining a "training set" of clinical data with approximately the same number of scans for each week of gestation, i.e. having scans in the "training set" uniformly distributed over gestational age (Altman and Chitty, 1994). However, this overlooks the fact that population data has a sharp peak of measurements around Week 12 (Supplementary Figure 6), and are thus far from uniformly distributed. This is due to the fact that population data are typically strongly selected to obtain an examination around Week 12, based on the LMP age. Since the "training set" and population data are selected very differently *on the variable that is to be predicted*, i.e. LMP gestational age, this approach may easily cause biased predictions (Økland et al., 2010). Similarly, this will impact prediction models fitted directly to population data, when LMP age is the target of the prediction. Our approach circumvents this issue altogether by first predicting remaining time, and then computing the implied gestational age. In our model, LMP age is only used to calibrate the total length of pregnancy; in our data this is found to be a median of 283 days. Supplementary Figures 7 and 8 illustrate the biases that appear when predicting LMP age at examination from the ultrasound measurements in population data.

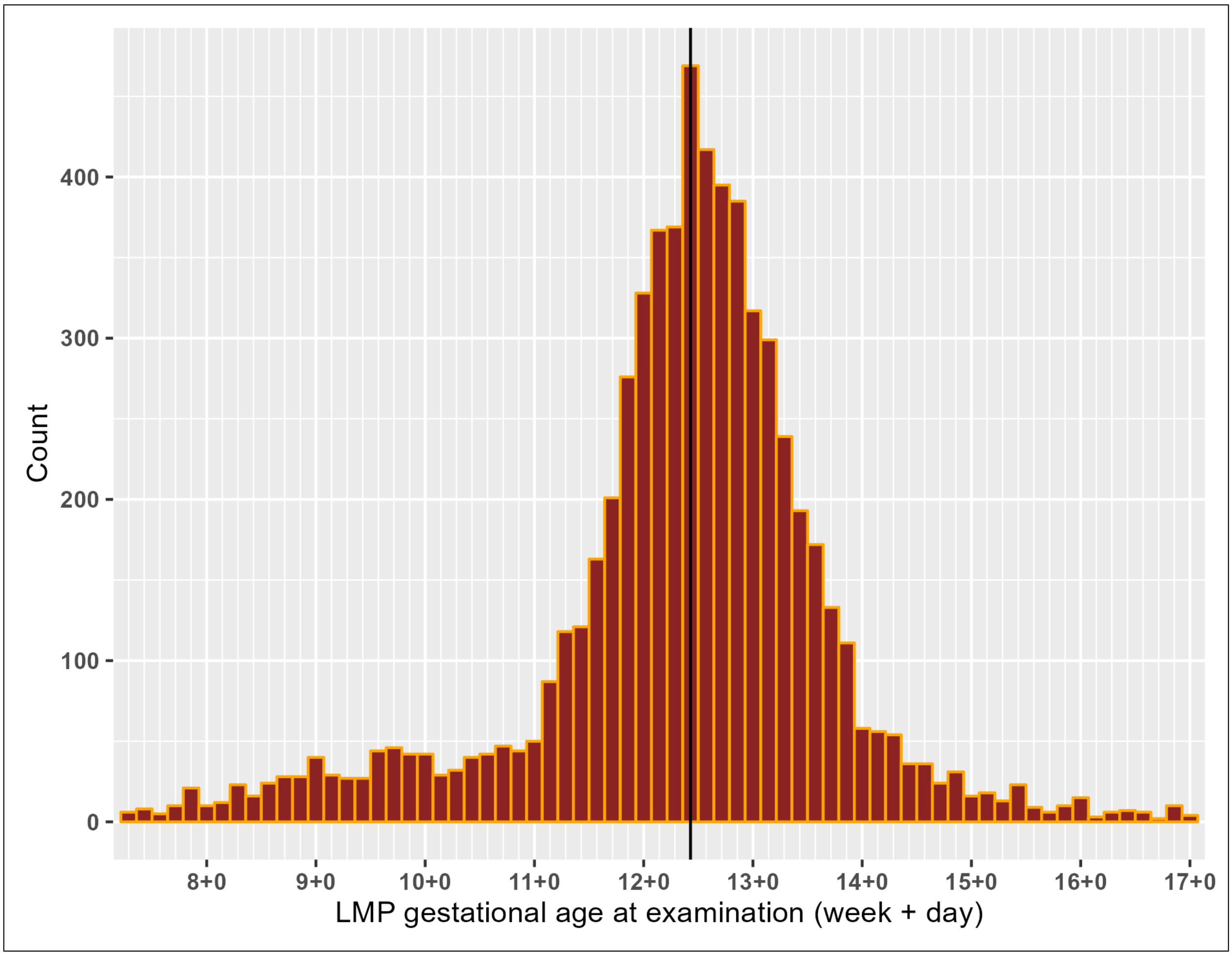

Supplementary Figure 6: *Bar chart of last menstrual period (LMP) gestational age at ultrasound examination. Black vertical line indicates median day of examination. Based on 12 260 examinations with either CRL or BPD in the appropriate ranges.*

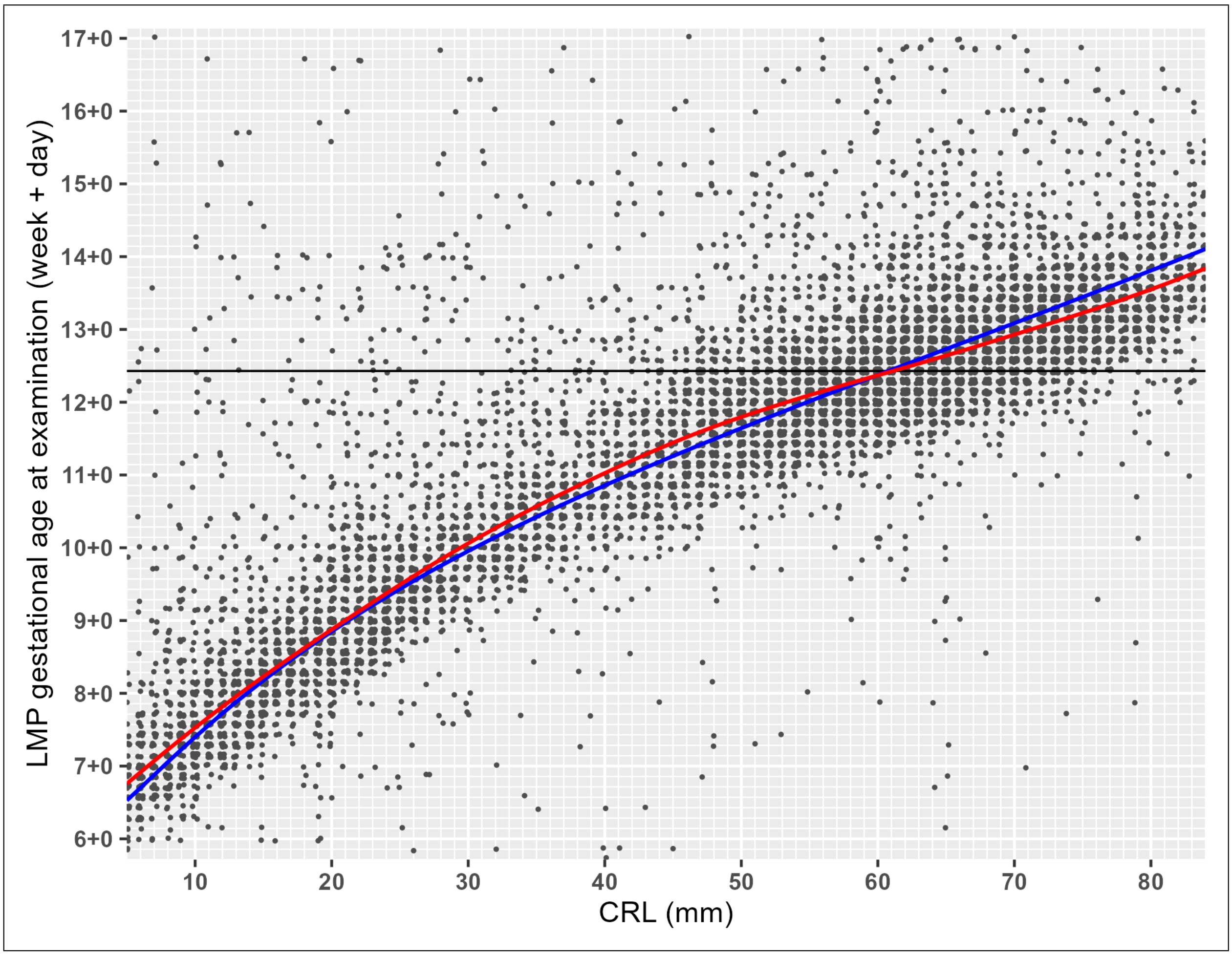

Supplementary Figure 7: *Scatterplot of last menstrual period (LMP) gestational age versus crown-rump length (CRL). Red line: Median LMP gestational age for each millimeter of CRL (slightly smoothed). Blue line: Gestational age prediction from CRL, based on the LLQRC model described in the main paper. Black horisontal line: Gestational age 12+3. Based on 11 651 examinations with CRL in the range 5 mm to 84 mm.*

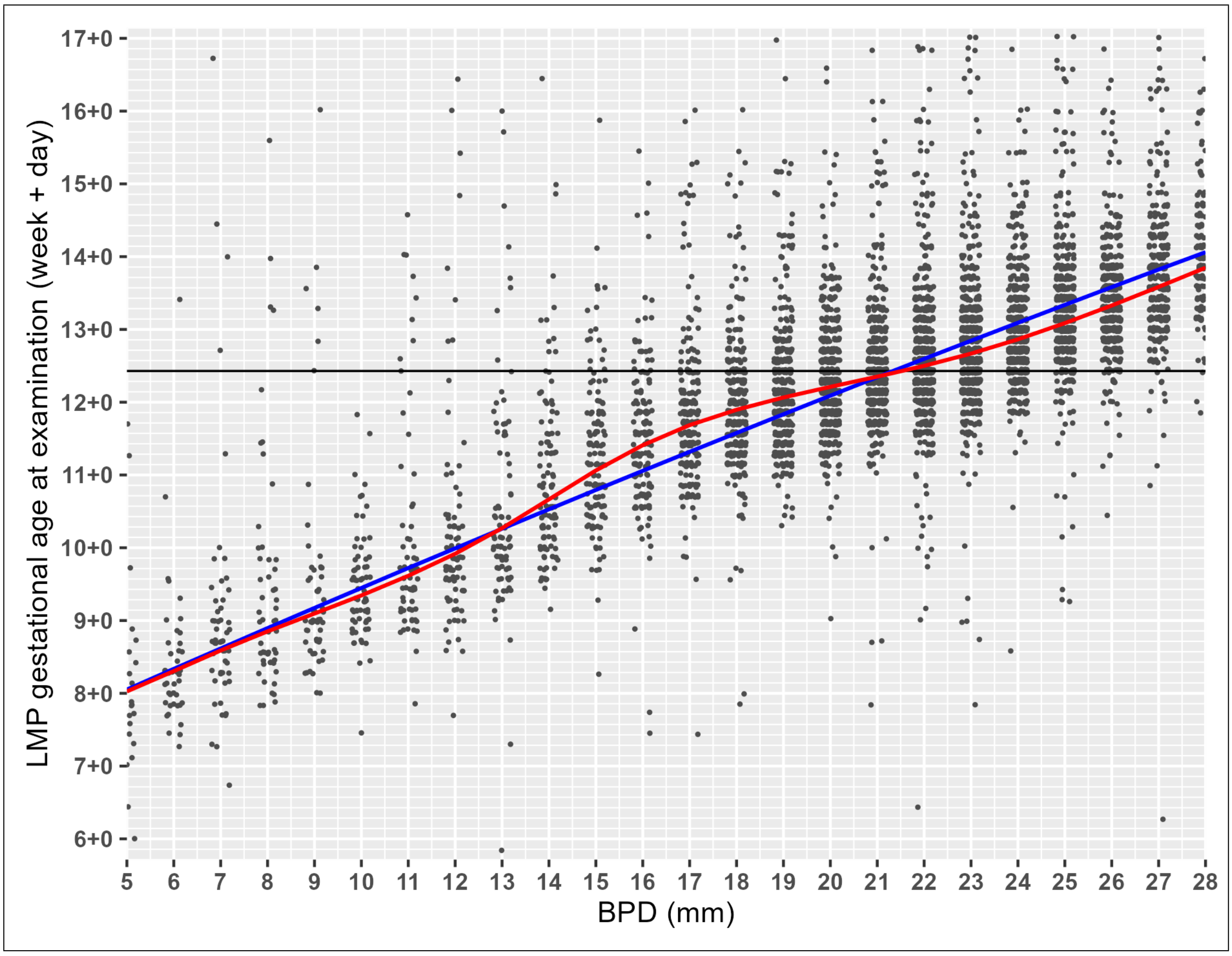

Supplementary Figure 8: *Scatterplot of last menstrual period (LMP) gestational age versus biparietal diameter (BPD). Red line: Median LMP gestational age for each millimeter of BPD (slightly smoothed). Blue line: Gestational age prediction from BPD, based on the LLQRC model described in the main paper. Black horisontal line: Gestational age 12+3. Based on 8 161 examinations with BPD in the range 5 mm to 28 mm.*

Supplementary Figure 6 shows how the distribution of LMP age at examination is concentrated around the median value of 12+3. In Supplementary Figures 7 and 8, the red line is computed simply as the median LMP gestational age for each CRL or BPD millimeter value, and slightly smoothed. It is clearly seen that the red line "gravitates" towards the 12+3 LMP median age, creating a wave pattern in the predictions. The blue lines, computed from the LLQRC model, are not directly impacted by this selection.

References

Altman, Douglas G. and Lyn S. Chitty (1994), "Charts of fetal size: 1. Methodology." *BJOG*, 101, 29–34, URL `http://onlinelibrary.wiley.com/doi/10.1111/j.1471-0528.1994.tb13006.x/abstract`.

Gjessing, H. K., P. Grøttum, and S. H. Eik-Nes (2007), "A direct method for ultrasound prediction of day of delivery: a new, population-based approach." *Ultrasound in Obstetrics and Gynecology*, 30, 19–27.

Hastie, Trevor, Jerome Friedman, and Robert Tibshirani (2001), *The Elements of Statistical Learning*. Springer Series in Statistics, Springer New York, New York, NY, URL `http://link.springer.com/10.1007/978-0-387-21606-5`.

Koenker, Roger (2023), “quantreg: Quantile Regression.” URL `https://CRAN.R-project.org/package=quantreg`.

R Core Team (2022), *R: A Language and Environment for Statistical Computing.* R Foundation for Statistical Computing, Vienna, Austria, URL `http://www.R-project.org`.

Taipale, P and V Hiilesmaa (2001), “Predicting delivery date by ultrasound and last menstrual period in early gestation.” *Obstetrics and Gynecology*, 97, 189–194.

Yu, K. and M. C. Jones (1998), “Local Linear Quantile Regression.” *Journal of the American Statistical Association*, 93, 228.

Økland, I, H K Gjessing, P Grøttum, and S H Eik-Nes (2010), “Biases of traditional term prediction models: results from different sample-based models evaluated on 41 343 ultrasound examinations.” *Ultrasound in Obstetrics & Gynecology*, 36, 728–734.
